# Genome-wide identification and expression analysis of MIKC^C^ genes in rose provide insight into their effects on flower development

**DOI:** 10.3389/fpls.2022.1059925

**Published:** 2022-11-02

**Authors:** Yi Wang, Tuo Yang, Yuqi Li, Jialin Hou, Junna He, Nan Ma, Xiaofeng Zhou

**Affiliations:** State Key Laboratory of Agrobiotechnology, Beijing Key Laboratory of Development and Quality Control of Ornamental Crops, Department of Ornamental Horticulture, College of Horticulture, China Agricultural University, Beijing, China

**Keywords:** MIKC^C^, gene family, rose, expression analysis, low temperature

## Abstract

The MIKC^C^-type gene family plays important roles in plant growth, development, and tolerance of biotic and abiotic stress, especially during floral organ differentiation. However, there have been no studies of MIKC^C^-type genes in rose, and functional differentiation of family members has not been explored. In this study, we identified 42 MIKC^C^-type genes in rose, classified the genes into 12 subfamilies, and constructed a phylogenetic tree. We performed expression analysis of these genes, and found that expression patterns correlated with the predicted subfamily, indicating that the features of MIKC^C^-type genes were broadly conserved during evolution. Collinear analysis of MIKC^C^ genes among Rosaceae species confirmed the occurrence of whole genome duplications (WGD) and revealed some species-specific MIKC^C^ genes. Transcriptome analysis showed that the expression of some MIKC^C^-type genes responded to low temperatures (4°C, 24 h) during flower organ differentiation. These conserved, duplicated, and novel expression patterns of MIKC^C^-type genes may have facilitated the adaptation of rose to various internal and external environmental changes. The results of this study provide a theoretical basis for future functional analysis of the MIKC^C^ genes in rose and investigation of the evolutionary pattern of the MIKC^C^ gene family in the Rosaceae genome.

## Introduction

The MADS-box transcription factor family is one of the largest families of transcription factors, and its members play critical roles during plant growth and development, especially in the development of root, flower, and fruit (Shore and Sharrocks, 1995; Causier et al., 2010). The MADS family was named for four DNA-binding proteins: MCMI (*Saccharomyces*), AGAMOUS (AG) (*Arabidopsis*), DEFICIENS (*Antirrhinum majus*), and SERUM RESPONSE FACTOR (SRF) (*Homo sapiens*) (Sommer et al., 1990; Alvarez-Buylla et al., 2000). MADS-box family genes can be divided into type I and type II classes. The type I MADS-box genes contain ARG80/SRF-like genes (animals and fungi) and diverse MADS-box genes (plants), which lack a ‘K’ domain and are categorized into Mα, Mβ, and Mγ classes (Günter et al., 1996; Parřenicová et al., 2003). The type II MADS-box genes include MEF2-like genes (animals and fungi) and MIKC-type genes (plants) (Alvarez-Buylla et al., 2000). MIKC-type proteins are conserved in structure, with a highly conserved DNA-binding MADS (M) domain, an intervening (I) domain, a keratin-like (K) domain, and a C-terminal (C) domain (Günter et al., 1996). The I domain is crucial to the formation of DNA dimers, the K domain is involved in protein-protein interaction *via* a coiled-coil structure, and the C domain is required for transcriptional activation and ternary complex formation (Wang et al., 2020). MIKC genes can be further classified into two categories, MIKC^C^ and MIKC*. During the evolution of land plants, a gene duplication event occurred in the MADS-box genes; MIKC^C^-type proteins retained the original domain structure, while MIKC*-type proteins acquired an extended ‘I’ domain (Becker and Theißen, 2003; Kaufmann et al., 2005). MIKC^C^-type genes have been phylogenetically and functionally characterized in various plant systems, including Arabidopsis, rice, wheat, and litchi (Becker and Theißen, 2003; Arora et al., 2007; Schilling et al., 2019; Guan et al., 2021). MIKC^C^-type genes play vital roles in flowering time, floral organ characteristics, and fruit maturity in plants, especially during floral transition and development (Theissen and Melzer, 2007; Li et al., 2016; Barrero-Gil et al., 2021).

The ABCDE model was established by analysis of homeotic floral mutants. Class A and E genes specify the identity of sepals; Class A, B, and E genes specify the identity of petals; class B, C, and E genes specify the identity of stamens; class C and E genes specify the identity of carpels; class D and E genes specify the identity of ovules (Causier et al., 2010; Silva et al., 2016). In angiosperms, class A genes include *SQUAMOSA/APETALA1* (*AP1*) and *APETALA2* (*AP2*); class B genes include *APETALA3* (*AP3*) and *PISTILATA* (*PI*); class C genes include *AGAMOUS* (*AG*); class D genes include *SHATTERPROOF* (*SHP*) and *SEEDSTICK/AGAMOUS-LIKE11* (*STK*); class E genes include *SEPALLATA1*, *2*, *3*, *4* (*SEP1*, *2*, *3*, *4*) (Wang et al., 2020). All the genes in the ABCDE model are MADS-box family members except *AP2*. MIKC^C^-type genes are also involved in the regulation of floral transition. Previous studies found that *SUPPRESSOR OF OVEREXPRESSION OF CONSTANS 1* (*SOC1*) regulates flowering time and flower initiation (Barrero-Gil et al., 2021); FLOWERING LOCUS C (FLC) targets *SQUAMOSA PROMOTER BINDING PROTEIN-LIKE 15* (*SPL15*) and *SHORT VEGETATIVE PHASE 1* (*SVP1*) to produce different flowering phenotypes and regulate floral transition mechanisms (Madrid et al., 2020); *AGL17* is a key regulator of floral transition (Shu et al., 2020). MIKC^C^-type transcription factors play crucial roles in regulating gene expression under various abiotic stress conditions (Saha et al., 2015; Guo et al., 2016; Wang et al., 2018; Chen et al., 2019). In *Brassica rapa*, 19 *BrMADS* genes were analyzed, and expression of eight and six genes were induced by drought and salt stress, respectively. In *Solanum lycopersicum*, MADS-box protein SlMBP11 is a stress-responsive transcription factor and plays an important role in the positive regulation of salt stress (Saha et al., 2015; Guo et al., 2016). However, our understanding of the potential cold stress-related functions of MIKC^C^ transcription factors remains limited.

Currently, we have only a general understanding of the functions of MIKC^C^-type genes in rose from analysis of transcriptome data (Liu et al., 2018; Raymond et al., 2018). However, analysis based solely on transcriptome data can be limited by inaccurate mapping and incomplete characterization of MIKC^C^-type genes. The goal of this study was to better understand the evolution of MIKC^C^-type genes in Rosaceae and to facilitate future research on this important transcription factor family. The results of our work provide a detailed overview of the number, phylogeny, and expression of MIKC^C^-type genes in *Rosa chinensis*. We found that a large number of MIKC^C^ orthologous genes were retained during the differentiation of Rosaceae, and we speculate that the functions of these genes may be relatively conserved. Interestingly, MIKC^C^-type genes of rose showed novel expression patterns under low temperatures. Collinear analysis of MIKC^C^ genes among Rosaceae species (*Rosa chinensis*, *Fragaria vesca*, *Rubus chingii*, *Prunus persica*, *Pyrus pyrifolia*, and *Malus domestica*) confirmed the occurrence of whole genome duplications (WGD) and revealed species-specific MIKC^C^ genes. We hypothesized that these conserved, duplicated and novel expression patterns of MIKC^C^-type genes may have facilitated the adaptation of rose to various internal and external factors. The results of this study provide new insights into the functions of MIKC^C^ genes in rose, and provide the basis for future work to explore the evolution of Rosaceae.

## Materials and methods

### Plant materials and low temperature treatment of rose floral buds

Rose (*Rosa hybrida* cv ‘Samantha’) plants were cultivated and grown as described previously (Zhang et al., 2019). Briefly, rose plantlets were propagated by tissue culture. Rose shoots approximately 2 cm in length and with at least 1 axillary bud were used as explants and cultured on Murashige and Skoog (MS) medium supplemented with 1.0 mg/L 6-benzyl aminopurine (6-BA) and 0.05 mg/Lα-Naphthaleneacetic acid (NAA) for 30 days at 23°C, under a 16-h light/8-h dark photoperiod (Wu et al., 2016). Rose seedlings were then transferred to 1/2 MS medium supplemented with 0.1 mg/L NAA for 20-30 days for rooting. After rooting, plantlets were transferred to pots containing peat moss: vermiculite (1:1) and cultured at 23°C with a relative humidity of ~60% and 16-h light/8-h dark photoperiod. After the emergence of floral buds, the rose plants were incubated at 4°C for 24 h (low temperature treatment) (Lee et al., 2021).

### Genome-wide identification and phylogenetic analysis of MIKC^C^-type genes in *Rosa chinensis*

The Rosa chinensis OldBlush Hm r2.0 genome was downloaded from the Lipme database (https://lipm-browsers.toulouse.inra.fr/pub/RchiOBHm-V2/). The sequences of the previously identified MIKC^C^ genes of *Arabidopsis thaliana* (Becker and Theißen, 2003) were obtained and confirmed in the TAIR database (https://www.arabidopsis.org/). The HMM models of the SRF-TF (PF00319) and K-box (PF01486) were obtained from the Pfam database (http://pfam.xfam.org/). The MIKC^C^ candidate genes were screened using HMMER3 (E-value = 0.01) and BLASTP (E-value = 1e-5) software (Camacho et al., 2009; Finn et al., 2011). The sequences of the putative MIKC^C^ genes were confirmed to contain the complete MADS domain by searching the conserved domain database (CDD) (Lu et al., 2019) for further analysis. MADS genes in *Arabidopsis* were used to classify the RcMADS genes. Multiple sequence alignment of the full-length protein sequences was achieved using MUSCLE software with default parameters (Edgar, 2004). A phylogenetic tree of the AtMADS and RcMADS genes was subsequently generated using IQ-TREE 2 (Minh et al., 2020). The same strategy was used for the construction of the phylogenetic tree of the MIKC^C^ genes of Rosaceae.

### Conserved motif analysis of the RcMIKC^C^ genes

The full-length protein sequences of the RcMIKC^C^ genes were obtained and the conserved motifs were analyzed. To do this, we used the Multiple Em for Motif Elicitation (MEME) online program (v5.5.0; http://meme-suite.org) with the following parameters: the number of repetitions was set to zero or one and the maximum number of motifs was 20. The conserved motifs were visualized with TBtools software (Chen et al., 2020).

### Collinearity analysis and chromosomal location of RcMIKC^C^ genes

We downloaded the genome sequences and annotation information from the Genome Database for Rosaceae (GDR) (https://www.rosaceae.org/) containing strawberry, raspberry, peach, pear, and apple. The GFF annotation files of Rosaceae species were used to retrieve gene location information. Construction of orthologous gene collinearity maps was based on inferred differentiation times for Rosaceae species from previous studies (Xiang et al., 2016). Gene duplication and collinearity relationships were produced by default parameters using the multicollinearity scanning toolkit (MCScanX) (Wang et al., 2012), and these results were visualized by Circos (http://circos.ca/) with a minimum block size of 30.

The positions of RcMIKC^C^ genes on the choromosome were mapped using MapChart (https://www.wur.nl/en/show/Mapchart.htm).

### Transcriptome sequencing and differential expression analysis

A total of 1.5 µg RNA per sample was used as input material for rRNA removal, using the RiboZero rRNA Removal Kit (Epicentre, Madison, WI, USA). Five sequencing libraries (**Supplementary Table S1**) were generated using the NEBNext UltraTM Directional RNA Library Prep Kit for Illumina (NEB, Ipswich, MA, USA), according to the manufacturer’s recommendations. The libraries were sequenced on an Illumina NovaSeq 6000 platform (Illumina, San Diego, CA, USA), and paired-end reads were generated. Differential expression analysis of two groups of floral bud (three biological replicates per group) (**Supplementary Table S2**) was performed using the DESeq2 R package. Genes with an adjusted p-value < 0.05 and an absolute value of log_2_ (FoldChange) > 1, were designated as being differentially expressed. The transcriptome data for other tissues (L-leaves, FB-floral buds, UP-upper of petals, MP-middle of petals, BP-bottom of petals) were previously measured in our lab (not uploaded), and the transcript fragments per kilobase (FPKM) values are shown in **Supplementary Table S1**.

RhMIKC^C^ gene expression profiles in different tissues were converted to transcript fragments per kilobase (FPKM) in millions of mapped reads and the results are displayed as a heatmap using TBtools. Expression correlations between individual genes are also shown.

### RNA extraction and qRT-PCR

Samples were ground in liquid nitrogen, then Total RNA was extracted by RNA extraction kit (TaKaRa, Ohtsu, Japan). The quality of extracted RNA was measured by determining the 260/280 absorbance ratio in the range of 1.8-2.0, and the 260/230 absorbance ratio is more than or equal to 2.0. The purified RNA samples were reversely transcribed into cDNA using HiScript^®^ II Q RT SuperMix for qPCR (+gDNA wiper) (Vazyme, Nanjing, China) according to the manufacturer’s instructions. Next, qRT-PCR was conducted using the SYBR FAST qRT-PCR universal kit (mei5bio) in a Roche LightCycler 96 according to the manufacturer’s instructions. For each sample, qRT-PCR was performed with six biological replicates. *RhUBI2* was used as an internal control. The primers used in the experiments are shown in **Supplementary Table S3**. The data were analyzed by correcting to the signal for the internal control and the 2^–ΔΔCt^ method (Livak and Schmittgen, 2001).

### *In situ* hybridization

*In situ* hybridization method of floral bud tissue samples was as described previously (Ma et al., 2015; Zhang et al., 2019; Chen et al., 2021). *In situ* hybridization probes were synthesized by PCR amplification of the cDNAs of *RhAP1*, *RhFUL*, *RhMADS6-1*, *RhSOC1*, *RhAGL42-1*, and *RhAGL24-3* using gene-specific primers containing T7 and SP6 RNA polymerase binding sites, with T7 RNA polymerase binding site in the antisense probe and SP6 RNA polymerase binding site in the sense probe. The floral bud tissue samples were treated at 4°C and 23°C in a plant chamber, and then samples were fixed in 3.7% FAA overnight at 4°C, followed by gradient dehydration, embedding, sectioning, hybridizing, and observation by microscope.

### Gene structure and cis-acting element analyses of RcMIKC^C^ genes

The structure of the MIKC^C^ genes, including exons, introns, and UTRs, were obtained from the genome GFF3 file. The Gene Structure Display Server (http://gsds.gao-lab.org/) tool was used to visualize the gene structures. The cis-acting elements in the promoter of the RcMIKC^C^ genes (in the 2 kb sequences upstream from the start codon) were predicted using the PlantCARE (https://bioinformatics.psb.ugent.be/webtools/plantcare/html/). Finally, TBtools software was used to display the cis-acting elements on promoters (Chen et al., 2020).

### Interaction networks of MIKC^C^ proteins in rose

Orthologous genes in *Arabidopsis thaliana* corresponding to the RcMIKC^C^ genes were used to predict protein interaction networks. The protein sequences of AtMIKC^C^ genes were used to search in STRING 11.0 (https://string-db.org/).

### Statistical analysis

Statistical analysis was performed using GraphPad Prism 8.4.3 (GraphPad Software Inc., USA: http://www.graphpad.com/). Experimental data were analyzed using one-way analysis of variance (ANOVA) followed by Tukey’s multiple range test to compare different groups among the experimental sites at *P* < 0.05. Student’s *t*-test was used to assess differences between two sets of data. *, *P* < 0.05; **, *P* < 0.01; ***, *P* < 0.001; ****, *P* < 0.0001.

## Results

### Identification and conserved motif analysis of MIKC^C^ genes in *Rosa chinensis*

**Figure 1:**
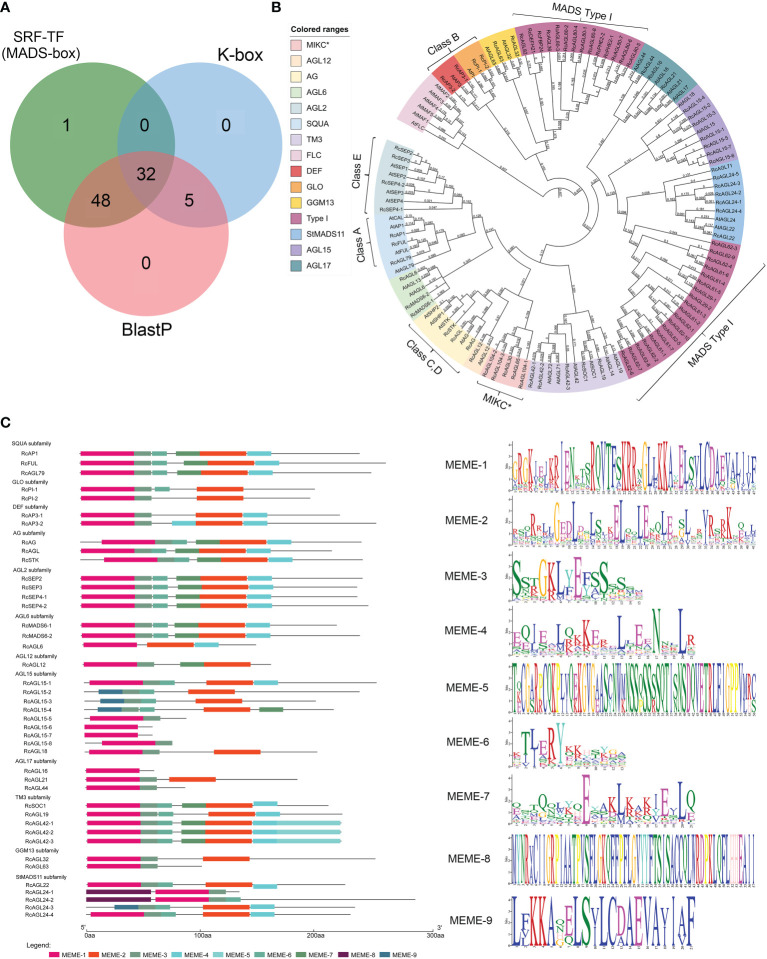
Identification of RcMIKC^C^ genes by HMM and BLASTP and analysis of their conserved domains. **(A)** Genes with MADS domains predicted by HMM and BLASTP. **(B)** Phylogenetic relationships among MADS genes in *Rosa chinensis* and *Arabidopsis thaliana.* **(C)** Analysis of the conserved domains of the RcMIKC^C^ genes identified by the phylogenetic tree.

The candidate genes with typical SRF-TF and K-box domain (PF00319 and PF01486) were preliminarily screened from the *Rosa chinensis* ‘Old Blush’ Hm r2.0 genome according to the Hidden Markov Model (HMM). The domain of MIKC^C^ is conserved, and the *Arabidopsis* MIKC^C^ genes were used as a reference to identify potential MIKC^C^ genes in rose. A total of 80 genes were initially identified in *Rosa chinensis*, as the overlap of HMM and BLASTP results (**Figure 1A**; **Supplementary Table S4**). The MADS domains of these genes were further classified by constructing a maximum-likelihood (ML) phylogenetic tree. We identified 42 conserved MIKC^C^ genes containing complete SRF-TF (MADS-box) domains in *Rosa chinensis*, including genes of the ABCDE model, flowering-related genes, and other genes (**Figure 1B**). The remaining 38 genes were clearly classified into MADS Type I (33 genes) and MIKC* (5 genes) families (**Figure 1B**). The MIKC^C^ genes distributed on seven chromosomes of rose, with two genes on chromosome 3 and fourteen genes on chromosome 7 (**Supplementary Table S5**). To further understand the characteristic motifs of the RcMIKC^C^ genes, Motif Elicitation (MEME) analysis was performed. The 42 MIKC^C^ genes contain nine motifs that are conserved in the same subfamily. Among them, The SQUA and AGL2 subfamilies contain six identical motifs; and the other subfamilies contain 1-7 motifs. MEME-1 and MEME-9 motifs had similar protein sequences. MEME-1 or MEME-9 motifs were found in all 42 RcMIKC^C^ genes, indicating high conservation of these motifs (**Figure 1C**). The MEME-5 motif is unique to the TM3 subfamily and is located at the end of the protein. Our analysis found that rose genes of the model share conserved domains, including MEME-1, MEME-2, and MEME-3. Class A, C, D, and E genes also contain the MEME-7 motif, which is not present in class B genes.

We also found five atypical MIKC^C^ genes that contained only the K-box domain. These were classified by constructing phylogenetic tree with the full-length protein sequences of the identified MIKC^C^ genes (**Supplementary Figure S1**). Subsequent analyses removed these atypical genes, and we focused on the MIKC^C^ genes with an intact SRF-TF (MADS-box) domain.

### Homology identification of MIKC^C^ genes in *Rosa chinensis*

**Figure 2:**
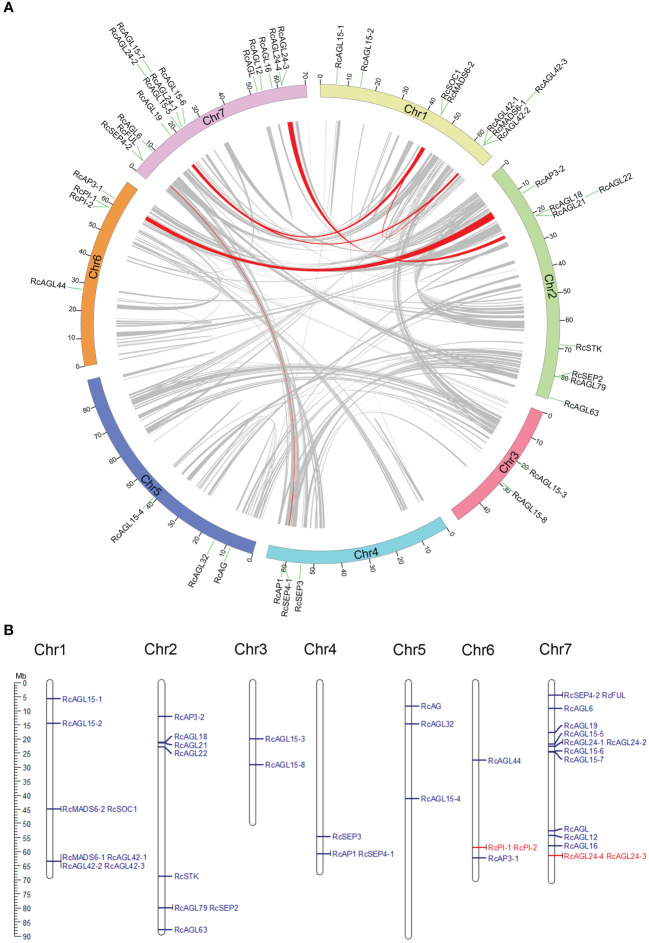
Identification of paralogous and tandem duplication genes of RcMIKC^C^. **(A)** Paralogous gene pairs in genome were analyzed by MCScanX and connected with red lines. **(B)** Karyotype diagram shows tandemly duplicated RcMIKC^C^ genes are marked in red.

Collinearity between MIKC^C^ genes was examined by MCScanX to identify paralogous genes. The potential collinearity results are shown in a circle plot, and the collinearity between genes is indicated with a red signal (**Figure 2A**). *RcAP1* is located on chromosome 4 (class A gene) of rose and has a paralogous gene *RcFUL* on chromosome 7. *RcAP3-1* is located on chromosome 6 (class B gene) of rose and has a paralogous gene, *RcAP3-2*, on chromosomes 2. *RcSEP4-1* (class E gene) on chromosomes 4 has a paralogous gene, *RcSEP4-2*, on chromosome 7. These highly similar class A, B, and C genes may be redundantly involved in the development of the four types of floral organs (sepals, petals, stamens, and carpels). There may be other paralogous genes, such as *RcMADS6-1* and *RcMADS6-2*, *RcSOC1* and *RcAGL42-1*, *RcAGL42-1* and *RcAGL19*, *RcSOC1* and *RcAGL19*, and *RcAGL22* and *RcAGL24-4*. The sequence similarity of these paralogous genes suggests they may have similar functions.

In *Rosa chinensis*, the number of singleton genes is 10,618, accounting for 24% of the total genes. The largest proportion of dispersed genes is 46%, and the number of tandem genes is 4,068, accounting for 9% (**Supplementary Figure S2**). Tandem duplications are multiple members of a family that occur in the same intergenic region. Tandem duplications are mainly caused by chromosomal recombination. In the 42 conserved MIKC^C^ genes of rose, *RcPI-1* and *RcPI-2*, and *RcAGL24-4* and *RcAGL24-3* resulted from tandem duplication as indicated by schematic representation of the chromosomal location (**Figure 2B**). The tandem duplication of these coding genes during evolution could generate novel paralogous genes with new or enhanced functions.

### Gene structure and promoter motif analysis in *Rosa chinensis*

**Figure 3:**
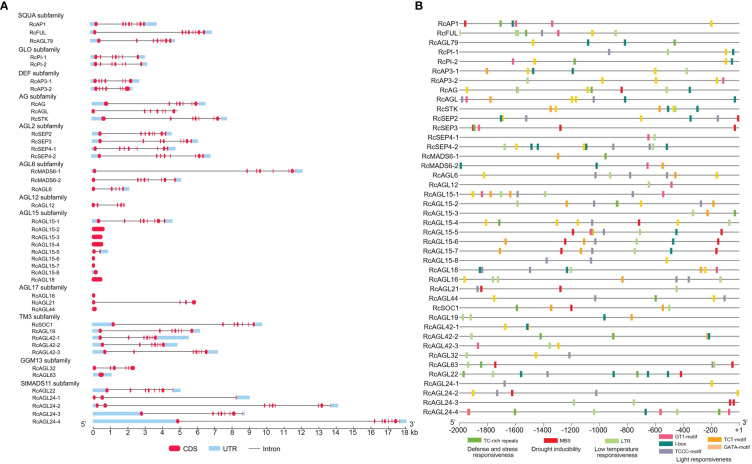
Gene structure and promoter response element analysis of RcMIKC^C^ genes. **(A)** Gene structure analysis of the RcMIKC^C^ family. The red labels, blue boxes, and black lines indicate exons, untranslated regions, and introns, respectively. **(B)** Analysis of hormone and stress response elements of the RcMIKC^C^ promoters.

To obtain a more comprehensive understanding of the structure of RcMIKC^C^ genes, we next analyzed the exon/intron organization of RcMIKC^C^ genes. Some of the RcMIKC^C^ genes have multiple exons, and the exons are distributed unevenly. AGL15, AGL17, and GGM13 subfamilies include genes with only one exon, and the AGL15 subfamily includes seven single-exon genes. The genes within the SQUA, AG and AGL2 subfamilies had eight exons and the genes within the GLO, DEF and TM3 subfamilies had 7 exons. The RcAGL6 and RcAGL12 genes had 5 exons. The number of exons in StMADS11 subfamily genes ranged from 3 to 9. The total gene length of RcAGL24-4 reached 18 kb, due to a large intron (**Figure 3A**). The varying gene structure indicated that RcMIKC^C^ genes may differ in many aspects, including gene function, evolution, mRNA process, and transcriptional regulation.

Significant regulation of gene expression can occur at transcription initiation with multiple regulatory elements in the promoter sequences. The general structure of a promoter includes core promoter elements and upstream regulatory elements. We extracted the promoter sequences of RcMIKC^C^ genes (2 kb) to search for potential regulatory motifs using the PlantCARE database (https://bioinformatics.psb.ugent.be/webtools/plantcare/html/). Many hormone-responsive elements were identified: auxin-responsive elements (AuxRR-core), SA-responsive elements (TCA elements), MeJA-responsive elements (CGTCA and TGACG motifs), gibberellin-responsive elements (TATC-box), and ABA-responsive elements (ABRE) were found in the promoter sequences (**Supplementary Table S6**; **Supplementary Figure S3**). Additionally, many abiotic response elements were identified, such as defense and stress-responsive elements (TC-rich repeats), drought-responsive elements (MBS), low temperature-responsive (LTR) elements, and light-responsive elements (GT1-motif; I-box; TCCC-motif; TCT-motif; GATA-motif). Of these, LTR elements were widely distributed in RcMIKC^C^ promoters (**Supplementary Table S6**; **Figure 3B**), so we inferred that low temperature may be an important signal regulating the expression of these genes.

### Transcriptional abundance and correlation of MIKC^C^ genes in *Rosa hybrida*

**Figure 4:**
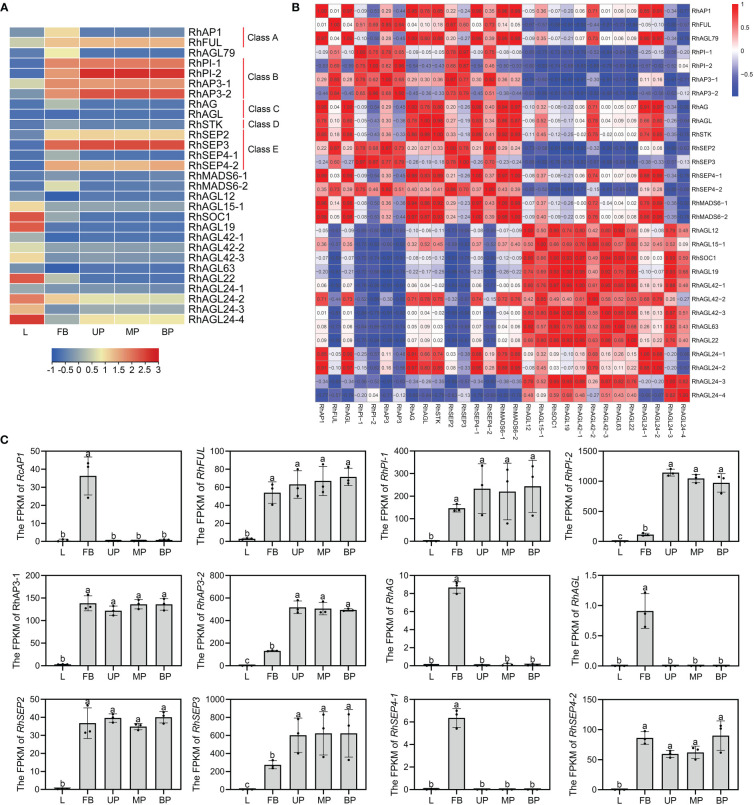
Expression patterns of RhMIKC^C^ genes by transcriptome analysis of rose leaves, floral buds, and petals. **(A)** Heatmap of RhMIKC^C^ gene expression in leaves (L), FB (floral buds), UP (upper of petals), MP (middle of petals), and BP (bottom of petals). Red color represents increasing gene expression and blue color indicates decreasing gene expression. **(B)** Expression correlation between individual genes is displayed in a heatmap. **(C)** The FPKM values of class ABCE genes in the transcriptome. Error bars indicate the standard error of the mean ± SD. Different letters above the bars indicate significantly different values (*P* < 0.05), calculated using one-way analysis of variance (ANOVA) followed by a Tukey’s multiple range test.

As one of the most important classes of MADS family, MIKC^C^ genes play vital roles in plant growth and flower organ development (Li et al., 2016; Barrero-Gil et al., 2021). To further investigate the differential expression of the MIKC^C^ genes in different tissues, transcriptome analysis was performed on different tissues (L-leaves, FB-floral buds, UP-upper of petals, MP-middle of petals, and BP-bottom of petals). The ABCDE genes were specifically expressed in floral buds or petals, consistent with their function of regulating flower organ development. The class A gene *RhAP1* was highly expressed in early floral buds, and another A class gene, *RhFUL*, was highly expressed in both floral buds and petals. There are two Class B genes, *PI* and *AP3*, in rose, and both were expressed in floral buds and petals. C genes *RhAG* and *RhAGL* were significantly expressed in early floral buds. The class D gene *RhSTK* exhibited greater transcript accumulation in floral buds compared to leaves and petals. As functionally redundant subfamily members (AGL2), class E genes are directly involved in multifaceted floral organ identity, and *RhSEP2*, *RhSEP3*, and *RhSEP4-2* were all detected in both early floral buds and petals. However, *RhSEP4-1* was highly expressed in floral buds, and not expressed in petals and leaves. *RhSOC1*, *RhAGL19*, *RhAGL22*, *RhAGL24-2*, and *RhAGL24-4* showed a trend of high expression in leaves (**Figure 4A**). Expression was not detected for additional 13 MIKC^C^ genes in leaves, floral buds, or petals. These included seven genes in the AGL15 subfamily, suggesting that these genes may not be directly involved in flower and leaf development. Gene ontology analysis of the 29 expressed MIKC^C^ genes indicated that 27 of them may have transcription factor activity and all may have protein dimerization activity (**Supplementary Figure S4**).

To study the expression correlation between MIKC^C^ genes, the expression matrix of the transcriptome was used as the original data to analyze the correlation (**Figure 4B**). In the SQUA subfamily, all genes except *RhFUL* showed a highly positive trend in expression. Class B genes and class C/D genes showed high positive correlation. Inconsistency in expression results in poor correlation of *RhSEP4-1* with other class E genes. There is a high positive correlation between the SQUA subfamily and the AG subfamily because the genes in these groups were expressed in floral buds. Genes among several subfamilies of AGL12, AGL15, TM3 and GGM13 were expressed in leaves, resulting in a positive correlation, as shown in the heatmap. The FPKM values of ABCE genes obtained from the transcriptome were used to visually display the expression trends in leaves, floral buds, and petals (**Figure 4C**). These genes showed low expression levels in leaves and high expression in floral buds, confirming the specificity of expression of these floral organ development genes.

### Expression patterns of MIKC^C^ genes in rose floral buds at low temperature

**Figure 5:**
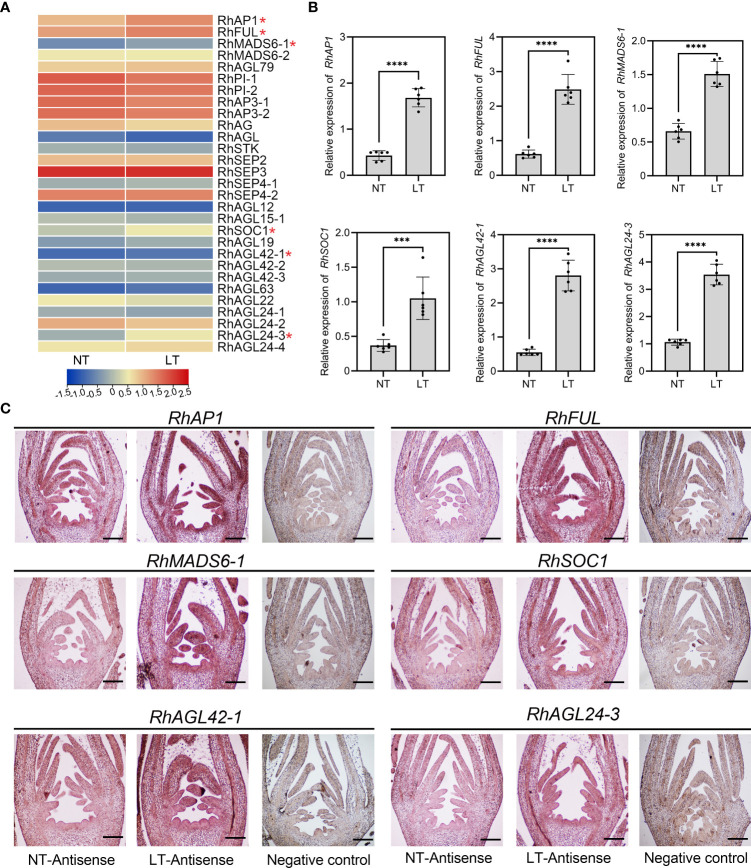
Transcriptome analysis of RhMIKC^C^ genes in rose floral buds at low temperature. **(A)** Heatmap of RhMIKC^C^ genes expression in transcriptome of floral buds treated with low temperature. Red color represents increasing level of gene expression and blue color indicates decreasing gene expression. **(B)** qRT-PCR analysis of the up-regulated RhMIKC^C^ genes expression in floral buds treated at 4°C for 24 h *RhUBI2* was used as an internal control. Asterisks indicate significant differences (two-sided Student’s *t*-test, ***P < 0.001, ****P < 0.0001). **(C)** *In situ* hybridization of the up-regulated RhMIKC^C^ genes in floral buds under normal temperature and low temperature (4°C for 24 h) floral buds. The sense probe was used as a negative control. Images show vertical sections of floral buds. Scale bar, 200 µm.

Low temperature usually results in flower malformation (Ma et al., 2015), which seriously compromises ornamental quality and economic value. Our analysis revealed that LTR elements are widely distributed in promoters of the RcMIKC^C^ genes (**Supplementary Table S6**; **Figure 3B**). Therefore, we next investigated the effect of low temperature on floral bud differentiation. Many genes regulating floral organ development are MIKC^C^ genes, so we focused on their expression after 24 h of treatment at 4°C by heatmap analysis (**Figure 5A**; **Supplementary Table S2**). *RhAP1*, *RhFUL*, *RhMADS6-1*, *RhSOC1*, *RhAGL42-1*, and *RhAGL24-3* were significantly up-regulated under low temperature. These six differentially expressed genes were further tested qRT-PCR and *in situ* hybridization to validate the accuracy of the RNA-Seq data (**Figures 5B, C**). Surprisingly, the expression of *RhMADS6-1*, *RhSOC1*, *RhAGL42-1*, and *RhAGL24-3* were not detected in floral buds at normal temperature, but increased expression was detected at low temperature. These results suggest that the MIKC^C^ genes of rose induced by low temperature may be involved in the development of flower organs under short-term low temperature stress.

### Interaction network analysis of the MIKC^C^ proteins in rose

**Figure 6:**
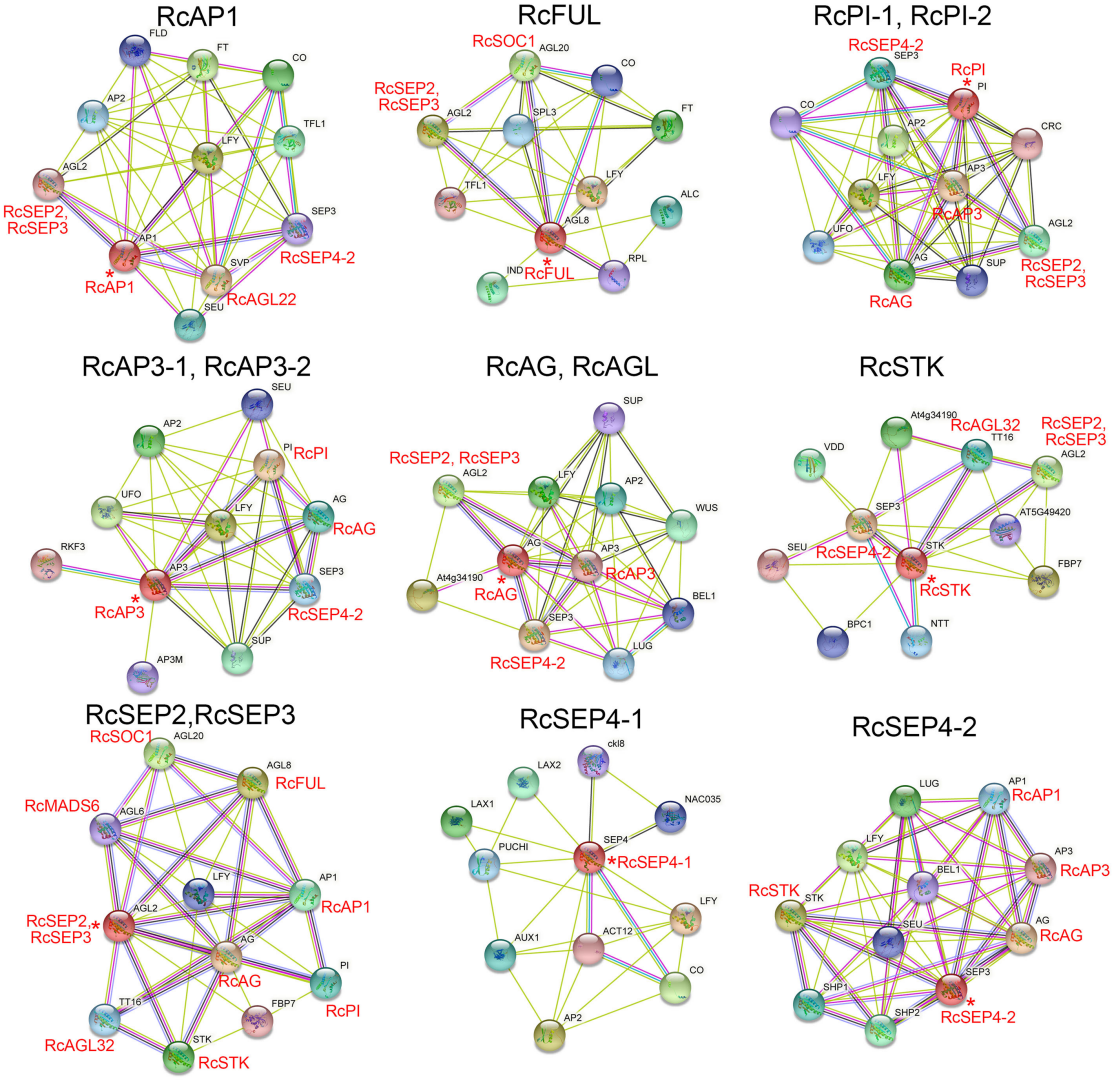
Interaction networks of RcMIKC^C^ proteins according to orthologs in *Arabidopsis*. Asterisks (*) represent core genes in the interaction network.

Previous studies reported that MADS proteins can form protein dimers (Kaufmann et al., 2005). The moderately conserved K-box domain of MADS proteins is important for protein-protein interactions and promotes the formation of α-helical structures. The orthologous genes of *Arabidopsis thaliana* were used to construct the RcMIKC^C^ protein interaction network (**Figure 6**; **Supplementary Table S7**). In the constructed network, class A genes (RcAP1 and RcFUL), class B genes (RcPI and RcAP3), class C genes (RcAG and RcAGL), and class D genes (RcSTK) may interact with the class E genes, respectively. Additionally, RcAP1 may interact with RcAGL22, and RcFUL may interact with RcSOC1. Interestingly, RcPI may interact with RcAP3, and RcPI and RcAP3 may both interact with RcAG. RcSTK may interact with RcAGL32. RcSEP2 and RcSEP3 may interact with RcSOC1, RcMADS6, and RcAGL32 (**Figure 6**; **Supplementary Table S7**). These protein interactions may provide clues to the novel functions of RhMIKC^C^ genes.

### Identification and collinearity analysis of MIKC^C^ genes in rosaceae

**Figure 7:**
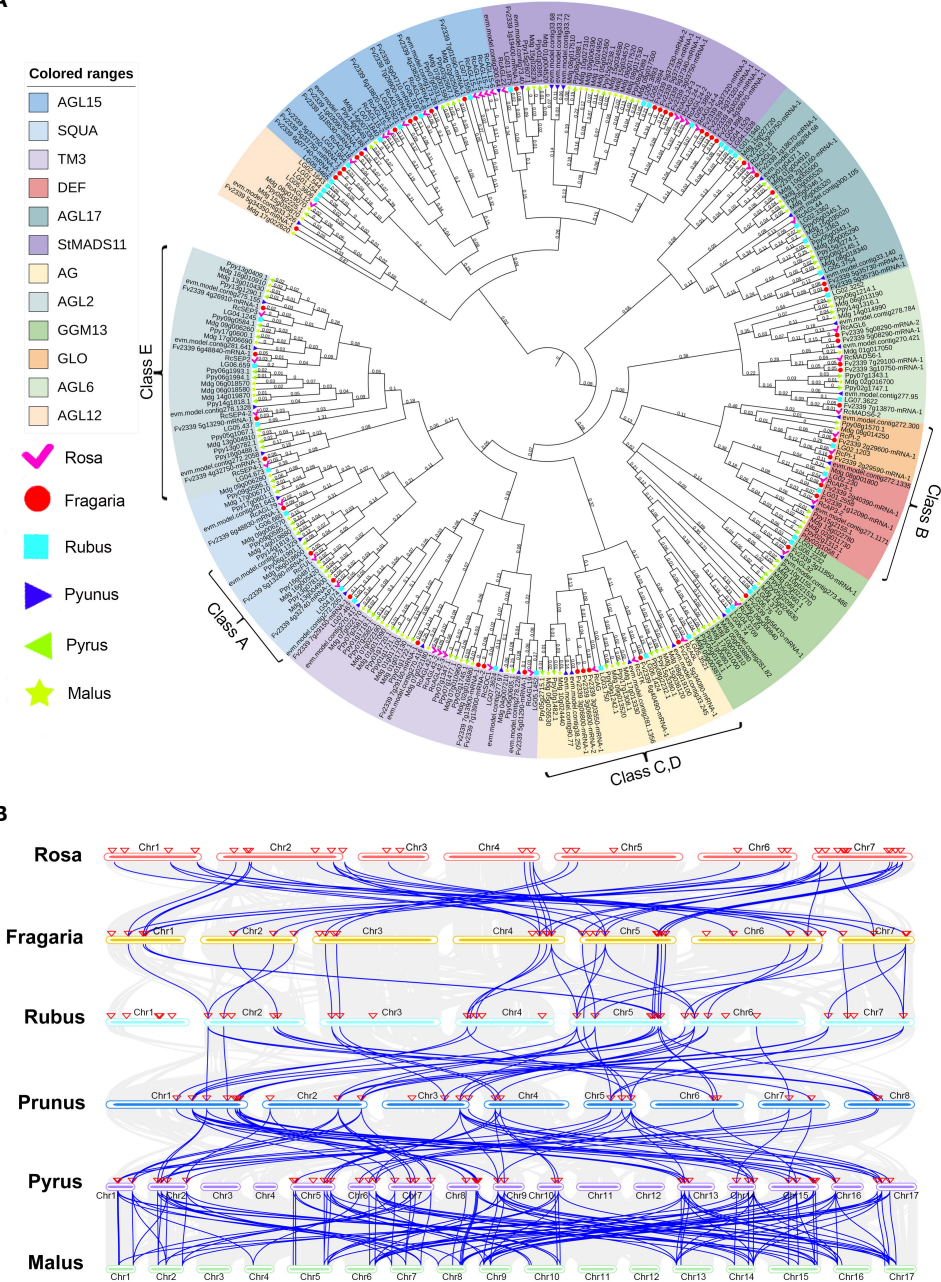
Identification of orthologous genes of MIKC^C^ in Rosaceae. **(A)** Phylogenetic tree of MIKC^C^ genes in Rosaceae. The MIKC^C^ gene members of rose (*Rosa chinensis*), strawberry (*Fragaria vesca*), raspberry (*Rubus chingii*), peach (*Prunus persica*), pear (*Pyrus pyrifolia*), and apple (*Malus domestica*) were analyzed separately. **(B)** Collinear analysis of MIKC^C^ genes among six Rosaceae species. The blue lines represent the collinearity between species, and the red triangles indicate the location of the MIKC^C^ genes.

The Rosaceae family is widespread worldwide, with more than 3,000 species, three subfamilies, 16 tribes, and 88-100 genera (Xiang et al., 2016). As a member of the Rosaceae family, rose is an important ornamental crop. To explore the evolution of Rosaceae MIKC^C^ genes, rose (*Rosa chinensis*), strawberry (*Fragaria vesca*), raspberry (*Rubus chingii*), peach (*Prunus persica*), pear (*Pyrus pyrifolia*), and apple (*Malus domestica*) were selected as comparative genomes, and the sequences of the MIKC^C^ genes in these genomes were retrieved. All the identified genes were combined for multiple sequence alignment, and an ML phylogenetic tree was constructed to classify the MIKC^C^ genes of each species. A total of 306 MADS genes were used to construct this phylogenetic tree (**Figure 7A**), and 13 subfamilies of MIKC^C^ were definitively identified (FLC subfamily genes are not present in Rosaceae). By comparison of Rosaceae species, the orthologous genes of MIKC^C^ with complete SRF-TF domains were effectively identified. We found that there were differences in the number of MIKC^C^ identified in the different species, and there are significantly more MIKC^C^ genes in pear and apple (**Supplementary Table S8**; **Supplementary Figure S6**). Both pear and apple experienced whole genome duplications (WGD) in the process of Rosaceae differentiation, resulting in multiple paralogous genes for many genes in the genome, and the number of chromosomes is increased to 17.

The order of colinearity analysis is arranged according to the time of differentiation of Rosaceae and revealed that some MIKC^C^ genes are conserved in Rosaceae. *RcAGL15-1*, *RcMADS6-2*, *RcSOC1*, *RcMADS6-1*, *RcAP3-2*, *RcAGL21*, *RcAGL22*, *RcSTK*, *RcAGL79*, *RcSEP2*, *RcSEP3*, *RcAP1*, *RcAG*, *RcAGL32*, *RcSEP4-2*, *RcFUL*, *RcAGL19*, *RcAG,L* and *RcAGL24-4* are conserved in Rosaceae (**Supplementary Table S9**). These conserved MIKC^C^ genes may be directly involved in plant growth and floral organ development (**Figure 7B**). We found *RcAGL42-3*, *RcPI-2*, *RcAGL15-5*, *RcAGL24-1*, *RcAGL24-2*, *RcAGL15-6*, and *RcAGL15-7* only present in rose, with no homologous genes in strawberry, raspberry, peach, pear, and apple genomes (**Supplementary Table S9**). These genes in rose may have contributed to the unique traits of this plant, and also suggest that rose may have multiple evolutionary branches.

## Discussion

MIKC^C^ genes play important roles in plant growth and developmental processes, as well as responses to biotic and abiotic resistance. In recent years, the expression of MIKC^C^ genes has been analyzed for many plants, such as *Solanum lycopersicum*, *Vitis vinifera* (Díaz-Riquelme et al., 2008), and *Rosa chinensis* (Liu et al., 2018). However, the evolutionary dynamics and functional analysis of the MIKC^C^ gene family in *Rosa chinensis* at the genomic level are still largely unknown. Here, a total of 42 MIKC^C^ genes were identified from the genome of *Rosa chinensis*, and these genes were assigned to 12 conserved subfamilies (but not the FLC subfamily) (**Figure 1**). There were similar total number of MIKC^C^ genes in different plants: *Rosa chinensis* (42), *Arabidopsis thaliana* (39), *Oryza sativa* (37) (Parřenicová et al., 2003; Arora et al., 2007), *Vitis vinifera* (32) (Díaz-Riquelme et al., 2008), and *Litchi chinensis* (37) (Guan et al., 2021). Almost all characterized MIKC^C^ genes are involved in flower transition and flower development (Causier et al., 2010; Smaczniak et al., 2012). We found that the AGL6, StMADS11 and AGL15 subfamilies in rose were significantly expanded from their corresponding clades in *Arabidopsis* (**Figure 1A**), suggesting that enhanced or novel roles of these genes might be required for specific aspects of floral transition and development in rose. In Wintersweet, AGL6 clade genes may positively regulate FT and negatively regulate FLC (Wang et al., 2011). In rice, members of the AGL17 clade may regulate flowering (Puig et al., 2013). In Arabidopsis, ALG15 clade genes may act in endosperm or gametophyte development (Adamczyk et al., 2007). AG, SQUA, AGL2, 406 DEF, and GLO subfamilies genes are specifically expressed in the reproductive organs (Causier et al., 2010; Smaczniak et al., 2012). Overall, the results suggest that MIKC^C^ genes play an extremely critical role in flower transition and flower organ development. Interestingly, there is no *FLOWERING LOCUS C* (*FLC*) and *MADS AFFECTING FLOWERING* (*MAF*) genes of the FLC subfamily in *Rosa chinensis*, as reported in previous studies (Liu et al., 2018; Raymond et al., 2018). The FLC subfamily genes play important roles in vernalization of some plants (Ratcliffe et al., 2001). Deletion of *FLC-like* genes in rose may result in a different flowering mechanism from Arabidopsis. Most modern roses have a continuous flowering trait, suggesting a novel regulatory mechanism.

Whole genome duplication (WGD) events are key drivers of gene evolution and expansion in many plants. These events can facilitate the emergence of new functional genes and species and make plants more tolerant to adverse environmental conditions (Cannon et al., 2003; Song et al., 2014). Previous results showed that MIKC^C^ genes are closely related to floral transition and floral organ development. According to the paralogous analysis of RcMIKC^C^ genes (**Figure 2**), both *RcPI* and *RcAP3* have paralogous genes, directly leading to the doubling of the B class genes in rose. Some RcMIKC^C^ genes were identified as tandem duplication events, such as *RcPI-1*, *RcPI-2*, *RcAGL24-3*, and *RcAGL24-4*, by co-linear analysis and chromosome localization. These results suggest that some RcMIKC^C^ genes may have emerged through gene duplication in *Rosa chinensis*, which further explains the mechanism of MIKC^C^ gene expansion. Collinearity analysis revealed differences in the duplication of MIKC^C^ genes (**Figure 2**), suggesting differences in retention and loss across subfamilies.

Phylogenetic analysis showed that RcMIKC^C^ genes can be divided into 12 subfamilies, and the five genes containing only the K-box domain were named *RcFUL-like*, *RcAGL6-like*, *RcAGL21-like*, *RcAGL24-3-like*, and *RcAGL24-4-like*. Since the predicted proteins lack the MADS domain, these are special MIKC^C^ genes (**Supplementary Figure S1**). The emergence of these special genes may be due to gene duplication in the distal telomeric segment, as previous studies have shown that gene duplication in the distal telomeric segment may lead to truncated genes lacking MADS or K-box domains. These sequences may encode proteins with impaired function (Schilling et al., 2019). Other examples were previously found, such as *TaSEP1-A2* (*WLHS1-A*), which lacks the ‘K’ domain and protein-protein interaction *in vivo* could not be detected (Shitsukawa et al., 2007). MIKC^C^ proteins lacking the K-box domain may theoretically be able to bind DNA and compete with other proteins for target sites, thus repressing transcription (Ferrario et al., 2004; Seo et al., 2012). The basic helix-loop-helix protein structure of the K-box domain may be important in the evolution and development of transcription factors (Jones, 2004). In this study, *RcFUL-like*, *RcAGL6-like*, *RcAGL21-like*, *RcAGL24-4-like*, and *RcAGL24-3-like* were identified with only the K-box domain may have new functions in the process of rose evolution. The diversity of sequences of these genes can help determine the evolutionary origin and functional relevance of RcMIKC^C^ gene duplication. Comparison of Rosaceae species reveals that MIKC^C^ genes in rose and strawberry are highly homologous, but raspberry lacks many of these homologous genes. There are no orthologous MIKC^C^ genes on chromosome 1 of raspberry, indicating that their production is independent.

Gene structure analysis revealed many MIKC^C^ genes with only one exon, and these are concentrated in the AGL15, AGL17, and GGM13 subfamilies (**Figure 3A**). The expression of these intron-less genes was not detected in leaves, floral buds, or petals of rose (**Figure 4**). In mammals, intron-less genes are expressed at lower levels, tend to be tissue specific, and evolve significantly faster than intron-containing genes (Shabalina et al., 2010). Intron-less genes often act in processes related to nucleosome assembly, signal transduction, and immunity (Louhichi et al., 2011). Introns increase the length of genes and the frequency of genetic recombination; although intron-less genes have no advantage in species evolution or genetic recombination, they can respond quickly to external abiotic stress (Shabalina et al., 2010; Fan et al., 2021). Currently, whether these rose MIKC^C^ family intron-less genes are functionally important remains an open question. We also found a gene, *RcAGL24-4*, 18 Kb in length due to a large intron insertion, which may explain its multiple paralogous genes. We classified 42 rose MIKC^C^ genes into five different categories according to the duplication type in the genome, namely singleton, dispersed, proximal, tandem, WGD or segmental (**Supplementary Figure S2**). Together, these results revealed that most rose genes have dispersed paralogous genes, indicating the high divergences of RcMIKC^C^ genes. This may be due to the functional diversity of gene families caused by chromosome rearrangement and fusion (Xu et al., 2012).

Low temperature an important environmental factor affecting floral transition and development (Acosta-Rangel et al., 2021; Jin et al., 2021). Therefore, we further investigated the effect of short-term low temperature on floral organ differentiation. We found that *RhAP1*, *RhFUL*, *RhMADS6-1*, *RhSOC1*, *RhAGL42-1*, and *RhAGL24-3* were significantly up-regulated under short-term low temperature treatment. Of all the RhMIKC^C^ genes, only significantly up-regulated genes were found, suggesting these genes respond to low temperature and regulate the development of floral organs. In *Arabidopsis*, the floral meristem recognition genes *AtAP1* and *AtLFY* are required for the transformation of inflorescence meristems into floral meristems (Gregis et al., 2008). Interestingly, *RhLFY* is also up-regulated in the low-temperature transcriptome data, and it regulates *RhAP1* expression as a pioneer transcription factor (**Supplementary Figure S5**). This may explain the absence of a low temperature response element (LTR) in the *RhAP1* promoter sequence. Some differentially expressed MIKC^C^ genes lack low temperature response elements, so differential expression may be due to the rapid cascade response of upstream transcription factors.

Previous studies have shown that *RhAG* (class C gene) expression was significantly reduced under low temperature treatment, promoting the stamen to petal transition (Zhang et al., 2019). However, the significantly inhibition of *RhAG* by low temperature was not detected in our results (**Figure 5A**; **Supplementary Figure S5**), which may reflect a difference in the treatment time or temperature. The up-regulation of class A genes (*RhAP1* and *RhFUL*) may play a key regulatory role in floral organ identity and development. Interestingly, a complex interaction network was constructed through orthologous genes among RhAP1, RhFUL, RhMADS6-1, RhSOC1, RhAGL42-1 and RhAGL24-3 (**Figure 6**). Dimerization between proteins can promote their ability to bind as transcription factors. These results provide a reference to explore the mechanisms related to floral organ differentiation and development under low temperature.

To understand in more detail the process of genome duplication in Rosaceae, we determined the number of MIKC^C^ genes in the AG, SQUA, AGL2, DEF and GLO subfamilies (ABCDE genes) of Rosaceae. Except for the B class, ACDE class genes showed significant gene duplication in *Pyrus* and *Malus* compared to the other Rosaceae species (*Rosa*, *Fragaria*, *Rubus*, and *Prunus*). This analysis indicated significantly increased numbers of MIKC^C^ genes in pear and apple. Peach, pear, and apple have experienced whole genome duplication events, and the collinearity relationship was observed from raspberry to peach with doubling of orthologous genes (**Figure 7B**). Although gene duplication occurred, the MIKC^C^ genes were highly conserved compared to rose and strawberry.

MIKC^C^ genes play central regulatory roles in flower transition and flower development in many plant species. Although MIKC^C^ genes have been studied for many years, the biological functions and regulatory mechanisms of these genes in the processes of flowering transition and floral organogenesis are not fully understood, especially in Rosaceae. Here, the MIKC^C^ genes in rose were comprehensively analyzed from gene structure, promoter elements, expression patterns and protein interaction to explore the roles of these genes in flower organogenesis and development in Rosaceae. This study provides a basis for marker development and targeted gene editing for future breeding efforts to enhance the ornamental value of rose.

## Data availability statement

The datasets presented in this study can be found in online repositories. The names of the repository/repositories and accession number(s) can be found below: https://www.ncbi.nlm.nih.gov/, SRR18440429-SRR18440424.

## Author contributions

XZ conceived and designed the experiments. YW, TY, YL and JlH performed the experiments and analyzed the data. JnH and NM provided technical support and conceptual advice. YW, TY and XZ wrote the article. All authors contributed to the article and approved the submitted version.

## Funding

This work was supported by the National Key Research and Development Program of China (Grant no. 2018YFD1000400) and National Natural Science Foundation of China (Grant no. 31972438).

## Conflict of interest

The authors declare that the research was conducted in the absence of any commercial or financial relationships that could be construed as a potential conflict of interest.

## Publisher’s note

All claims expressed in this article are solely those of the authors and do not necessarily represent those of their affiliated organizations, or those of the publisher, the editors and the reviewers. Any product that may be evaluated in this article, or claim that may be made by its manufacturer, is not guaranteed or endorsed by the publisher.

## Supplementary material

Supplementary Figure 1 Classification of K-box MIKC^C^ genes by phylogenetic tree. The full-length protein sequences of the RcMIKC^C^ genes were used to construct the phylogenetic tree using the maximum-likelihood method in IQ-TREE.

Supplementary Figure 2 Duplicated genes in rose.

Supplementary Figure 3 Gene structure and promoter response element analysis of RcMIKC^C^ genes.

Supplementary Figure 4 Gene Ontology molecular function annotation of RhMIKC^C^ genes identified in leaves, floral buds, and petals transcriptomes.

Supplementary Figure 5 The expression levels of *RhAG* and *RhLFY* under short-term low temperature stress.

Supplementary Figure 6 Statistics on the number of ABCDE genes in Rosaceae.
