# Supplementary figures and images for: Genome-wide identification and expression analysis of MIKCC genes in rose provide insight into their effects on flower development
Source: Front Plant Sci. 2022 Nov 2;13:1059925. doi: 10.3389/fpls.2022.1059925 (PMC9666904; doi:10.3389/fpls.2022.1059925)

**Figure S1**


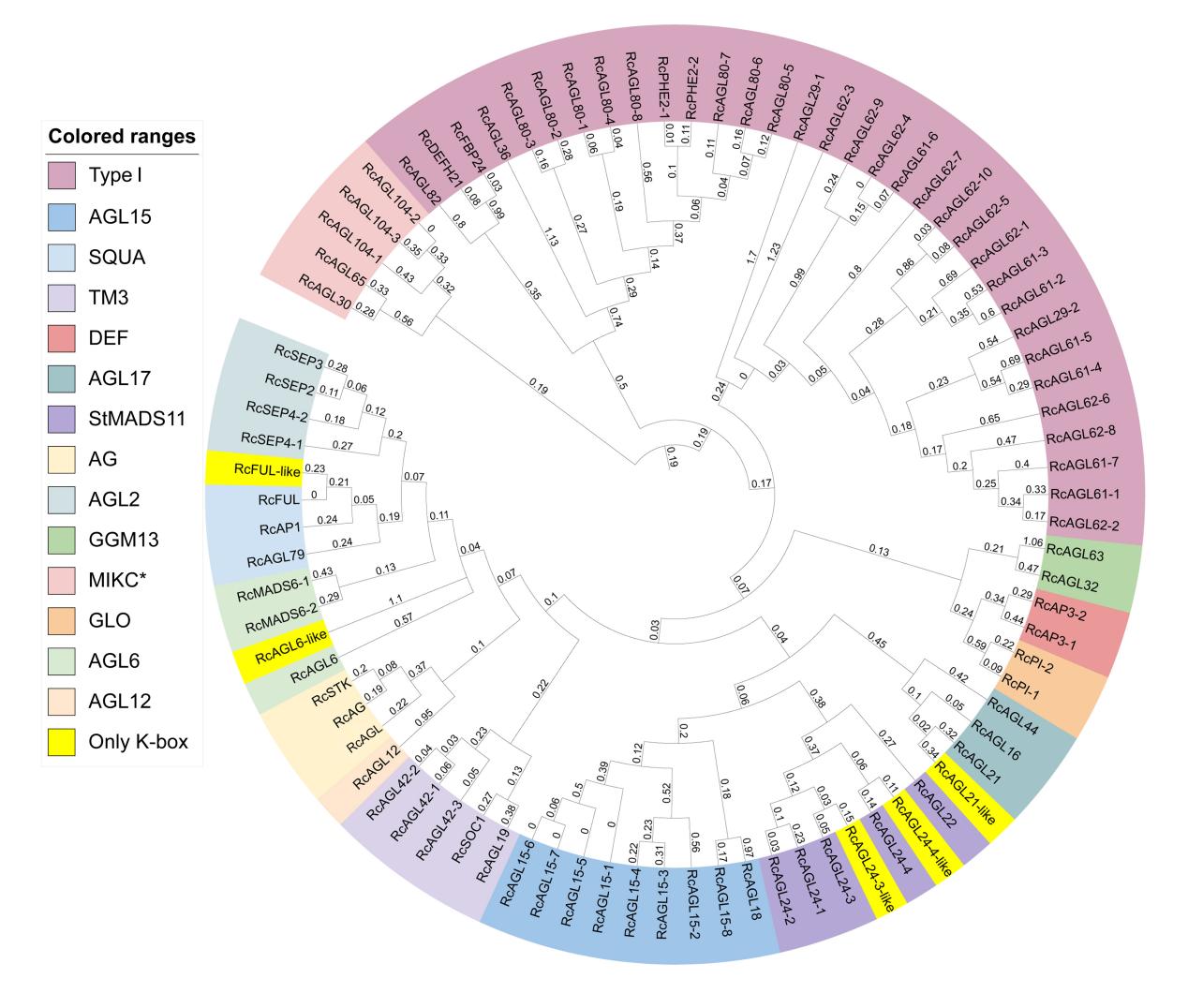


**Figure S2**

**
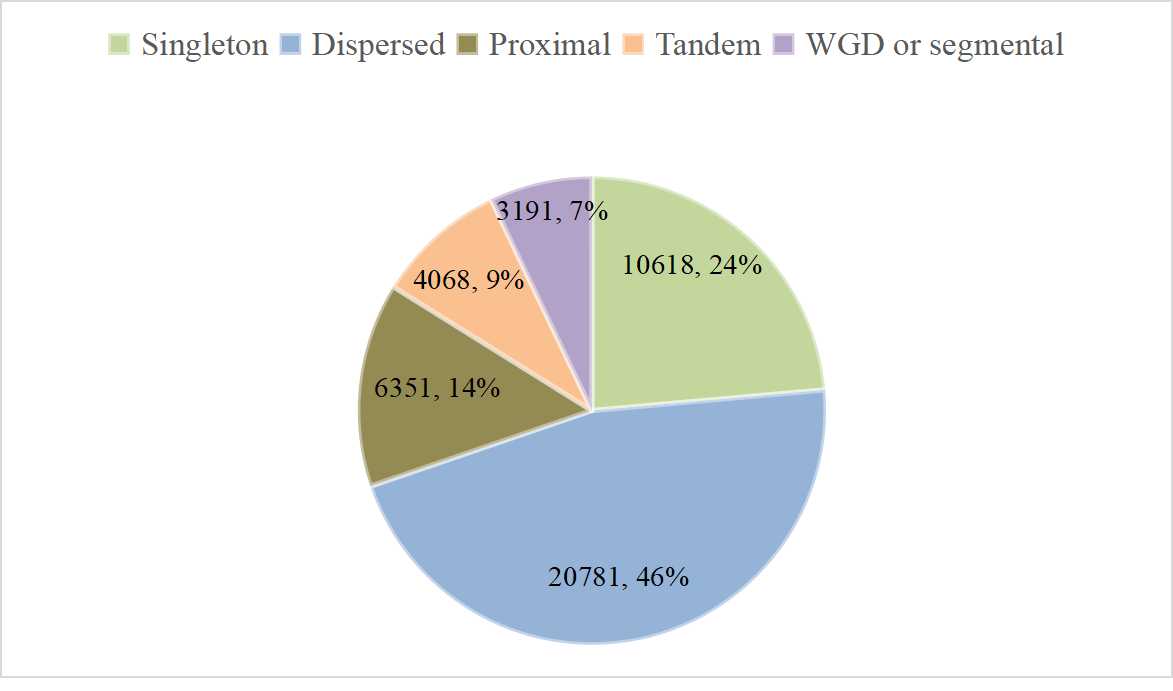
**

**Figure S3**

**
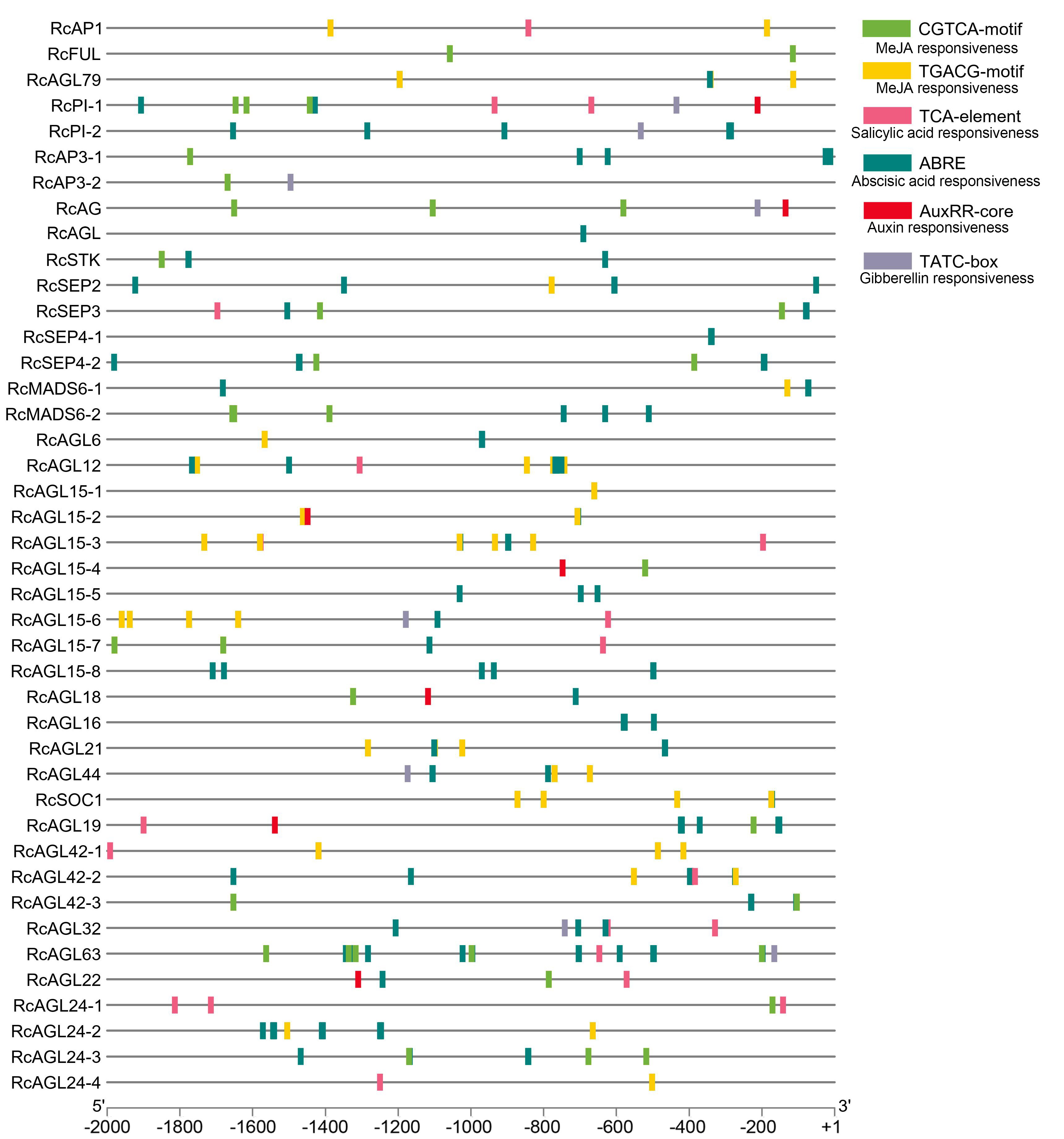
**

**Figure S4**


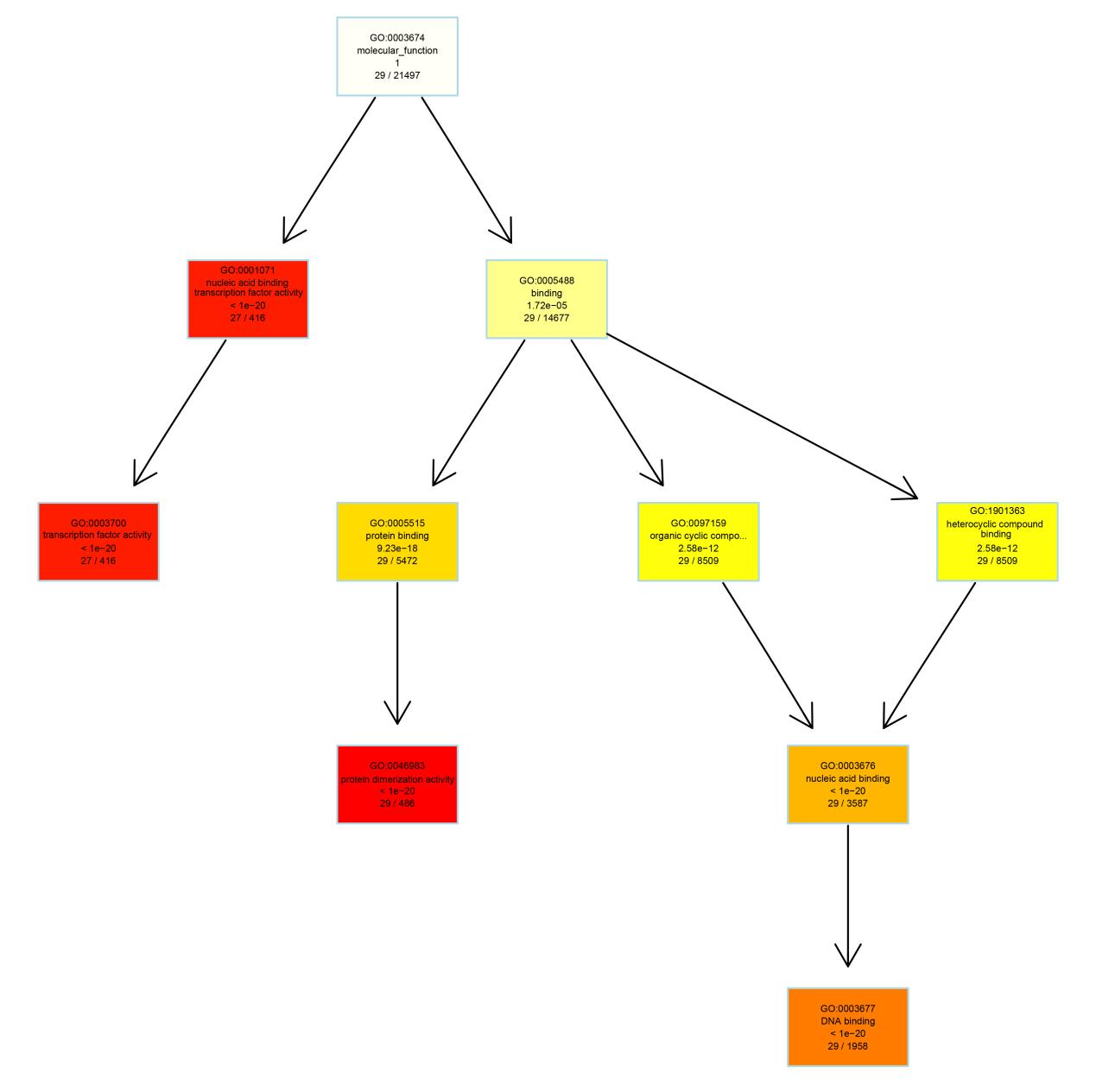


**Figure S5**

**
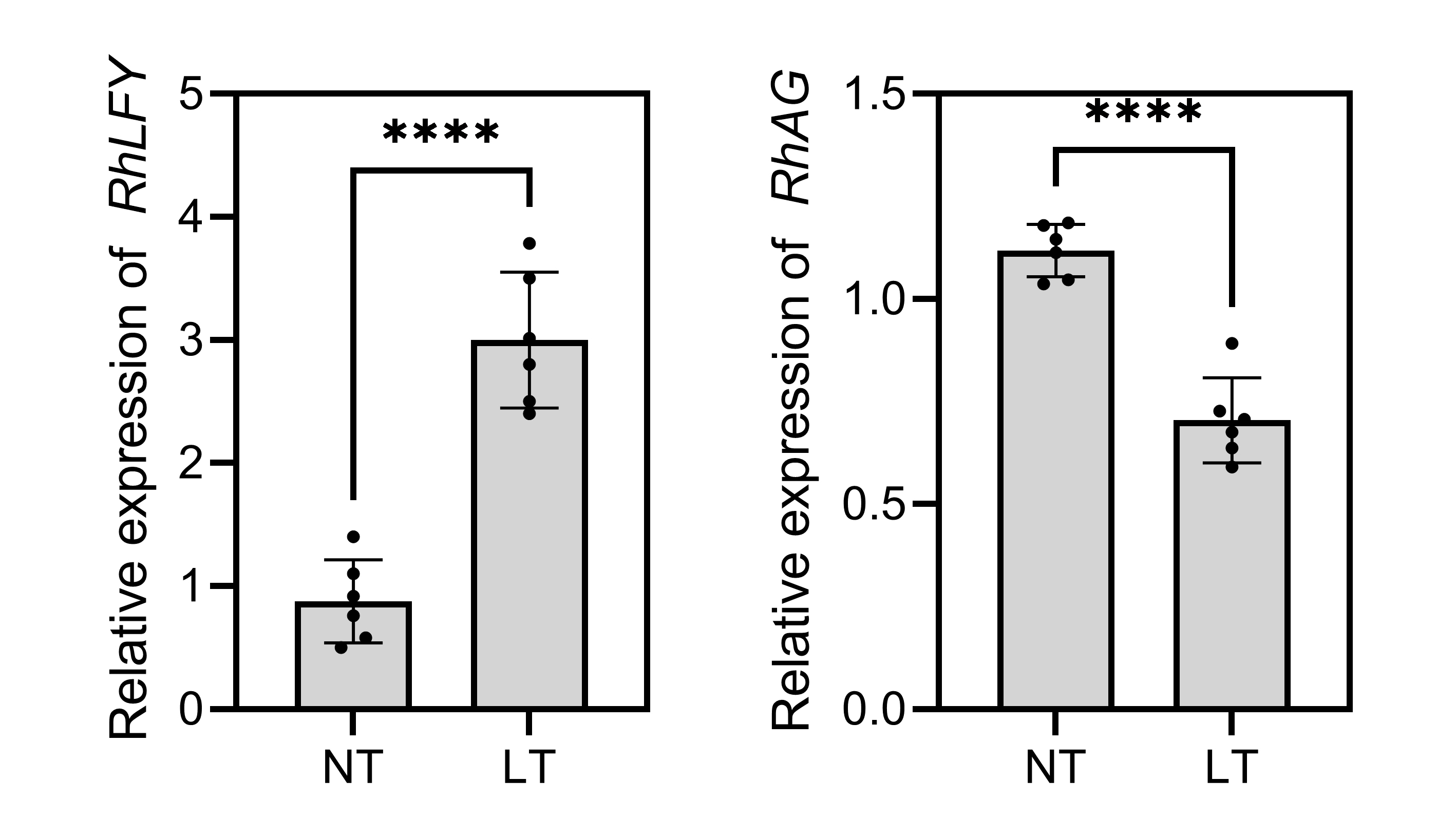
**

**Figure S6**


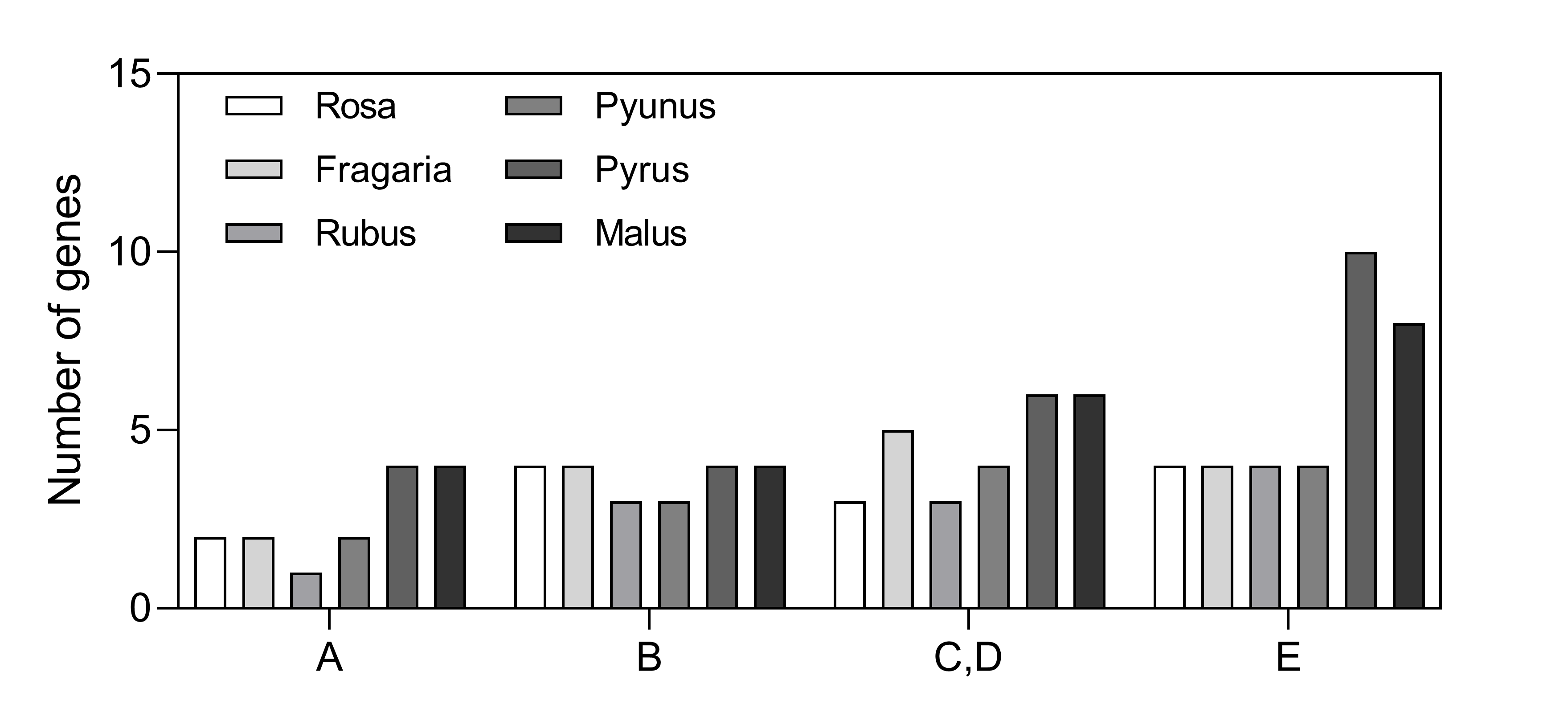

Supplement: Supplementary Figure 1 — Classification of K-box MIKCC genes by phylogenetic tree. The full-length protein sequences of the RcMIKCC genes were used to construct the phylogenetic tree using the maximum-likelihood method in IQ-TREE. [file DataSheet_1.docx]
